# Study on Hardness, Microstructure, Distribution of the Self-lubricating Phase, Friction and Wear Property of 1Cr13MoS after Heat Treatment

**DOI:** 10.3390/ma12193171

**Published:** 2019-09-27

**Authors:** Shaolong Li, Yusi Che, Jianxun Song, Chenyao Li, Yongchun Shu, Jilin He, Bin Yang

**Affiliations:** 1 Henan Province Industrial Technology Research Institute of Resources and Materials, Zhengzhou University, Zhengzhou 450001, China; lishaolong135@163.com (S.L.); cheys@zzu.edu.cn (Y.C.); lichenyao0329@163.com (C.L.); Shuyc@zzu.edu.cn (Y.S.); hejilin@zzu.edu.cn (J.H.); kgyb2005@126.com (B.Y.); 2 National Engineering Laboratory of Vacuum Metallurgy, Kunming University of Science and Technology, Kunming 650093, China

**Keywords:** 1Cr13MoS, self-lubricating, hardness, friction and wear performance

## Abstract

1Cr13MoS is a kind of material with excellent corrosion resistance and good mechanical properties. Meanwhile, it also has good self-lubricating properties due to the presence of molybdenum disulfide phase inside the material and can be used as friction pair material in the pump. In this paper, the hardness, microstructure, distribution of the self-lubricating phase, friction and wear properties of 1Cr13MoS after heat treatment were studied. After quenching at 1000 °C and tempering at 520 °C, the hardness of 1Cr13MoS prepared by pyrometallurgy is higher than that of HB 350. The tempering sorbite structure is evenly distributed, and the self-lubricating phase MoS_2_ is discretely distributed on the substrate with the average size is about 6 μm, which leads to good friction and wear properties. It is worth noting that the 1Cr13MoS is actually operated as friction pair material on the water pump and has a significant wear improvement effect compared to the conventional 12% chrome steel series.

## 1. Introduction

**Figure 1:**
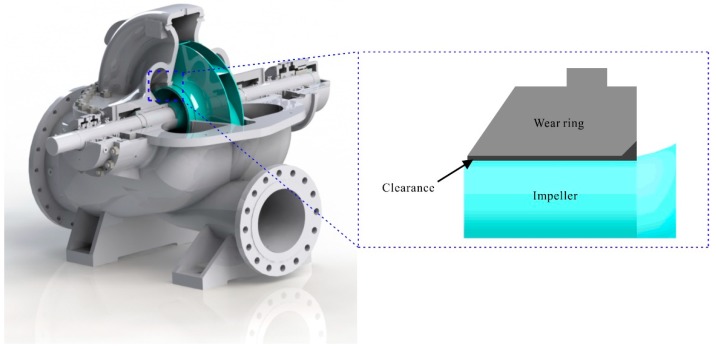
Schematic diagram of a certain type of water pump and impeller-wear ring friction pair.

12% chrome steel series is widely used for some key components in water pump such as impeller, guide vane, and wear ring due to its good heat resistance, cavitation resistance, and excellent mechanical properties [1,2]. As a rotor component, the impeller rotates at high speed during the pump operation. The wear ring acts as a fixed component and forms friction pair with the impeller as shown in Figure 1. There is a gap between the impeller and the wear ring. The size of this gap is generally determined according to experience or reference to relevant standards, which is generally between 0.25~0.60 mm. The oscillation of fluid and the vibration of pump shaft will make the wear ring come into contact with the impeller during the running of the pump, which will result in wear of the wear ring and the pump efficiency decreasing. In addition, due to the adhesive friction between the wear ring and the impeller during the starting and stopping process of the pump, the rotor parts and stator parts may be locked, thus damaging the pump. In order to avoid the above situations, the hardness of the wear ring generally needs to be HB 50 higher than the impeller [2]. The difference in hardness can be obtained by adjusting the chemical composition of the material or the heat treatment process parameters. However, in actual operation, the hardness difference of friction pair can not play an ideal role, and accidents caused by wear of the wear ring are still common, which greatly increases the risk of equipment operation and also increases the maintenance cost of the enterprises. In recent time, a number of companies are trying to change the material of the friction pair or add lubricating medium to reduce the probability of such accidents results from wear of the pump parts.

Since the impeller and the wear ring operate in a water environment, it is not possible to reduce the friction by applying a lubricating medium between them. Thus, a kind of self-lubricating material was applied for pump producing. It was reported that solid self-lubricating materials can extend the service life and have excellent lubrication performance under high temperature, heavy load, and high-pressure conditions [3,4]. MoS_2_ is one of the most commonly used solid lubricant in the world [5]. Based on stainless steel, a self-lubricating material containing MoS_2_ phase was studied, which has excellent anti-friction and wear properties. It is suitable for the case with oscillating motion, such as in the turbine [6,7,8,9].

Different from ordinary stainless steel, the material should be added with a certain proportion of sulfur and molybdenum on the basis of stainless steel to form a powder lubricant MoS_2_. After special heat treatment, the hardness of the material can reach above HB 350 with high wear resistance. The amount of S added needs to be strictly controlled. If the addition amount of S is too small, it cannot fully react with Mo to form MoS_2_ for good lubrication. If S is added too much, it will cause hot brittleness, reduce the mechanical properties of steel, and have a great adverse impact on the corrosion resistance and weldability of steel. Preparation techniques for sulfur-containing self-lubricating materials include powder metallurgy methods and conventional synthetic metallurgy methods [10,11,12]. The materials studied in this paper will be prepared using traditional smelting processes. In order to make the material a good comprehensive performance, it is necessary to make a reasonable blending of the chemical composition and heat treatment process of the material. In this paper, the self-lubricating martensitic stainless steel containing MoS_2_ phase was prepared by adding a certain amount of Mo and S into the basis of 12% chrome steel. Since there is no corresponding standard brand for this material, it is represented by 1Cr13MoS in this paper. Hardness, microstructure, self-lubricating phase, and friction properties of this material were studied.

## 2. Material and Method

### 2.1. Material Preparation

The 1Cr13MoS steel was smelted on the basis of 12% chrome steel series by reasonably controlling the chemical composition in a medium frequency furnace (Xi’an Kewen Mechanical and Electrical Equipment Co., Ltd., Xi’an, China). The main elements were added in the form of a simple substance except for S which was added as FeS. The casting temperature was 1530 °C. In terms of chemical composition control, the elements are similar to that of X12Cr13 (X12Cr13 is a material in the standard EN10088-3) [13] except S and Mo which can ensure that the strength and corrosion resistance of 1Cr13MoS material is similar to that of X12Cr13. The content of S is controlled at 0.25~0.32%. At the same time, Mo is added to generate MoS_2_ with S. In addition, Mn is also very easy to react with S to produce MnS. So the addition of Mn needs to be strictly controlled in order to reduce the generation of MnS. The material with this chemical composition can guarantee the strength and corrosion resistance, and at the same time, the material has self-lubricating properties. The chemical composition was controlled within the range demonstrated in Table 1. The chemical composition was analyzed in the smelting process, and the composition was regulated according to the analysis results. The chemical composition of the material measured after smelting is shown in Table 1, and all element contents are within the control range.

**Figure 2:**
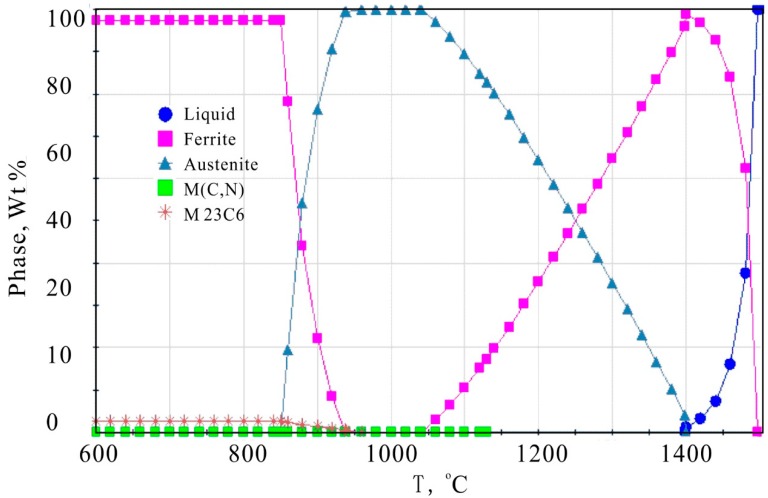
Phase diagram of 1Cr13MoS (the chemical composition is the measured value as shown in Table 1).

The heat treatment process of materials has a great impact on their mechanical properties. Figure 2 shows the phase diagram of 1Cr13MoS. As can be seen from Figure 2, the temperature at which the austenitization beginning is approximately 840 °C, and the finished temperature of austenitizing is approximately 910 °C. Thus, the quenching temperature was determined to be 1000 °C for making the material austenitized completely and not being overheated. In addition, the temperature of annealing and tempering for the material are determined to be 900 °C and 520 °C, respectively according to Figure 2 to ensure a uniform structure, uniform composition, and high hardness. It was quenched in oil, and the tempering cooling medium is air. The heating and holding time is determined according to the effective thickness of the sample. The temperature is controlled by a platinum-rhodium thermocouple with a temperature difference of 2 °C. This temperature difference has little effect on the quenched properties of the material.

### 2.2. Characterization of 1Cr13MoS

#### 2.2.1. Hardness and Microstructure Analysis

Since the stainless steel is heat-treated under air atmosphere, the carbon content of the surface layer will be reduced, forming a decarbonization layer, which causes changes of the surface microstructure, decreasing of hardness, declining in fatigue resistance, and process performance. In this paper, the depth of decarburization layer is determined by detecting the changes in carbon content and hardness of the surface layer. The specific steps are as follows: after heat treatment, the 1Cr13MoS sample was ground on the grinder (Shenyang Machine Tools. Ltd, Shenyang, China) and the grinding thickness is controlled at about 0.5 mm. Then the hardness and chemical composition of the sample was tested. The accurate grinding thickness was determined by micrometer (Shanghai Siwei Instrument Manufacturing Co., Ltd., Shanghai, China). Repeat the above procedure until the hardness and carbon content are stable. Finally, the microstructure of the cross-section was tested.

#### 2.2.2. Analysis of Self-Lubricating Phase and Impurity Phase

The Gibbs forming energy of MnS, FeS, and MoS_2_ were calculated, and the distribution of elements, especially Fe, Mn, Mo, and S, was analyzed by Energy Dispersive Spectrometer (EDS, Thermo Fisher Scientific, Waltham, MA, USA). The morphology and size of each sulfide phase were analyzed, and some impurity phases were also studied by electronic scanning microscope (SEM) and EDS.

#### 2.2.3. Friction and Wear Performance

**Figure 3:**
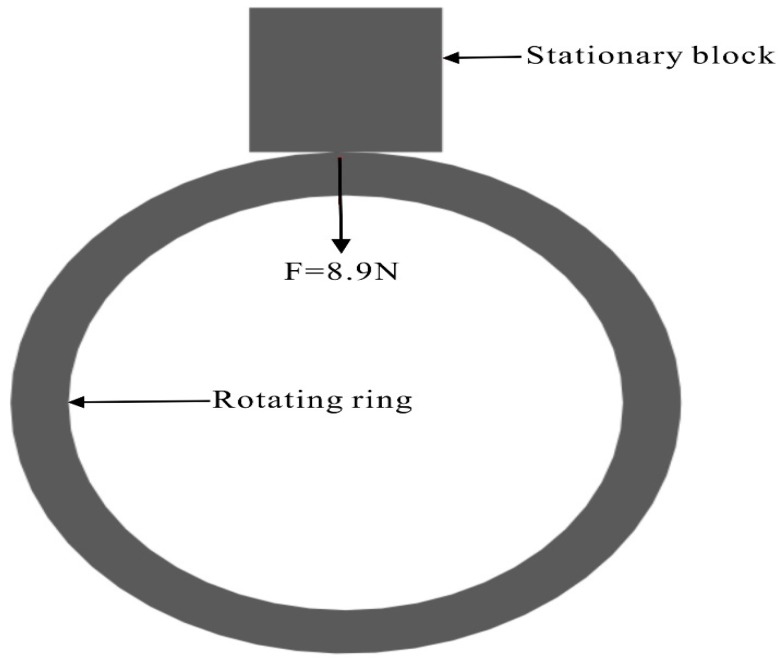
Schematic diagram of friction and wear experiment.

The friction and wear test were carried out on a block-on-ring tribometer, and the experimental diagram is shown in Figure 3. 1Cr3MoS and X20Cr13 (X20Cr13 is also a material in the standard EN10088-3) [13] with a hardness of HB 350 were used as a block (10 mm × 10 mm × 10 mm). The X12Cr13 with a hardness of HB 250 was processed into a ring for rotary motion (φ40 mm, external diameter × 10 mm, thickness). The surface roughness of the sample was controlled at Ra (μm)1.6. During the whole experiment, the friction pair was contacted directly without lubricant. The experimental load was 8.9 N, the rotational speed was 600 r/min, and the experimental time was 20 min. It should be noted that the friction pair model selected in this study is a ring-on-block, which is somewhat different from the ring-on-ring model in industrial applications. The two rings in industrial applications are concentric rings, and there is a certain gap between the rings, so they do not contact under ideal conditions. Ring to ring contact may occur under pump shaft vibration or water agitation. It is basically wear on a point or a plane. So, in this paper, the wear ring is reduced to a block, and the impeller is still a ring, which can restore the actual working condition at a certain level. Therefore, the ring-on-block model was selected in this study.

All the chemical components in this study were tested by a direct reading spectrometer (SPECTROLAB M8, Sylmar, CA, USA). The hardness test was performed using a Brinell hardness tester (HB-3000, Shanghai Test Instruments Co.,Ltd., Shanghai, China). Microstructure and friction surface morphology were observed by optical microscope (4XC) and SEM (Quanta 200, Houston, TX, USA). The friction test was performed using a block-on-ring tribometer (MRH-5A, Jinan Caide Instrument Co., Ltd., Jinan, China).

## 3. Results and Discussion

### 3.1. Hardness and Microstructure

**Figure 4:**
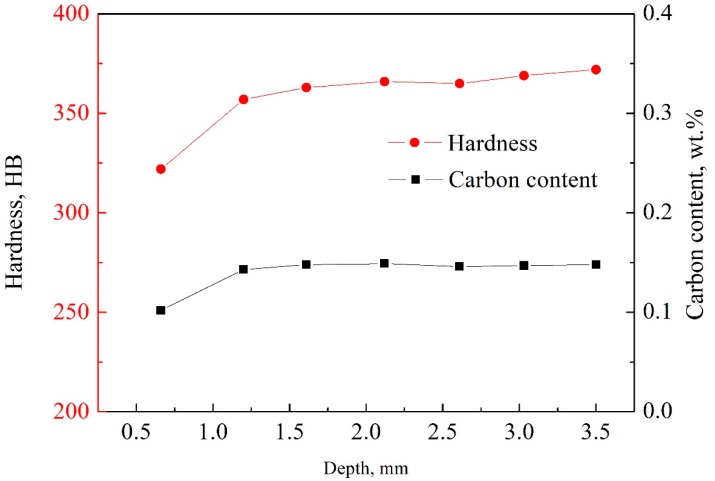
Curves of hardness and carbon content with depth.

After heat treatment, the carbon content of the surface layer of the sample changed, which resulted in the difference in hardness and microstructure between the surface layer and interior as shown in Figure 4. The hardness of the sample was measured by holding a 1 mm diameter cemented carbide pellet under a pressure of 0.294 kN for 12 s. The hardness of the steel range of HB 140-640 can be measured under this condition. The hardness is proportional to depth until the surface depth reaches 1.5 mm, and carbon content has the same trend. The hardness and carbon content tend to be stable when the depth is close to 1.5 mm, wherein the hardness reaches HB 360 and the carbon content is about 0.149%.

**Figure 5:**
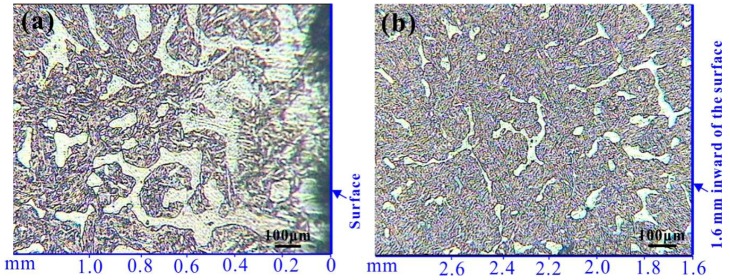
Microstructure of 1Cr13MoS obtained by optical microscope after heat treatment (after corrosion by ferric chloride hydrochloric acid solution): (**a**) surface, (**b**) internal.

Figure 5a shows the cross-section of the sample. As can be seen from Figure 5a, the white ferrite structure in the surface area is coarser, and the inner ferrite structure is smaller. The reason for the above phenomenon is that the ferrite phase grows rapidly due to the decrease of carbon content of surface layer during the quenching stage. Since the ferrite is soft, the hardness of the surface layer is lower than the inside. Figure 5b shows a relatively uniform microstructure of the inner region, which is gray tempered sorbite wrapped in some white ferrite with an irregular shape. Since the tempering temperature is only 520 °C, the tempered sorbite still has a martensitic orientation.

### 3.2. Analysis of Self-Lubricating Phase and Impurity Phase

#### 3.2.1. Distribution of Self-Lubricating Phase

**Figure 6:**
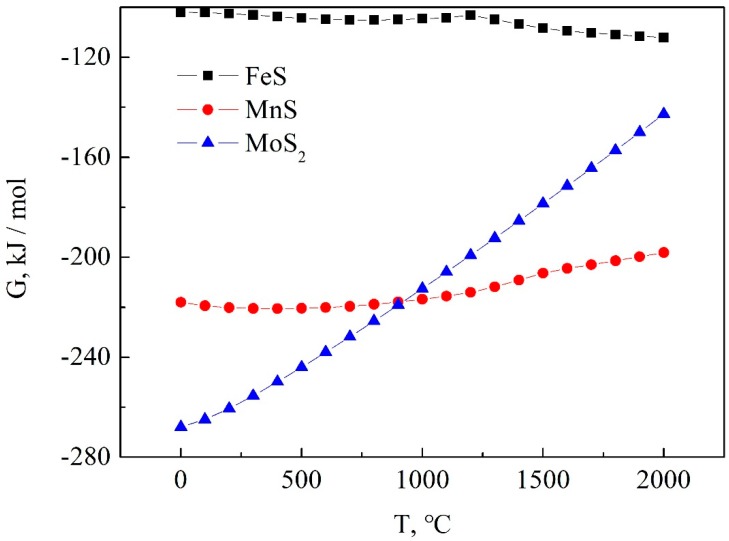
Gibbs formation energy of major sulfides in steel (the data of the Gibbs formation energy are obtained from HSC Chemistry).

Simple substance sulfur is easy to react with various metals to form compounds. The Gibss formation energy of MnS and MoS_2_ are higher than that of FeS as shown in Figure 6. MoS_2_ and MnS will be formed simultaneously in the whole smelting and casting process due to the similar formation of energy. Although MnS can also be used for lubrication, it is far less effective than MoS_2_. In addition, the MnS-steel matrix interface with its low bonding strength provides sites for hydrogen accumulation thereby increasing the susceptibility of the steel to hydrogen-induced cracking. Therefore, it is necessary to strictly control the amount of Mn during the smelting process to reduce the formation of MnS [14,15,16]. The content of Mn in steel can be controlled below 0.3 wt. %.

**Figure 7:**
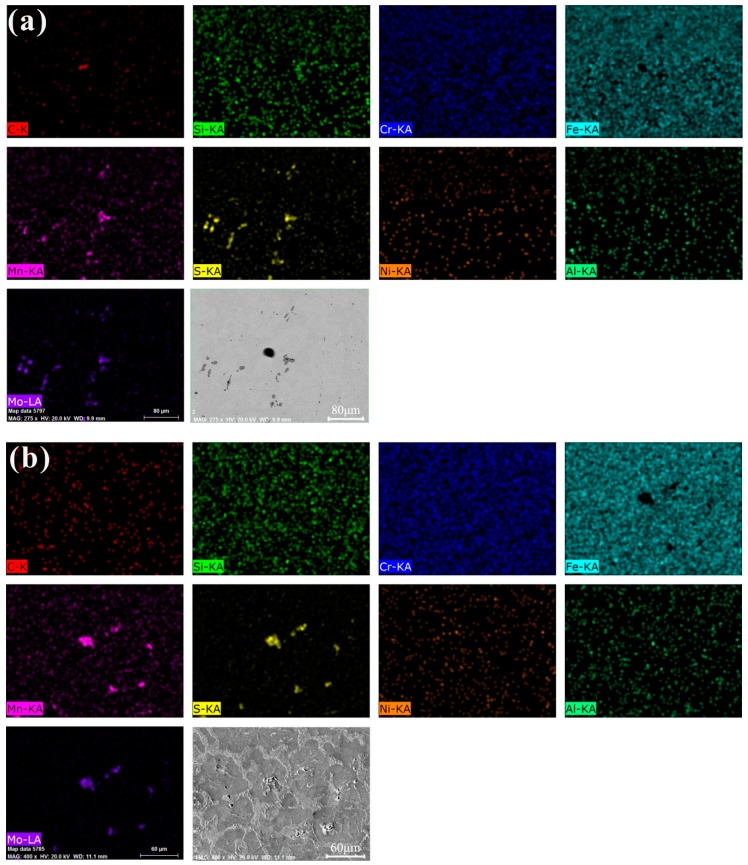
Distribution of chemical elements in 1Cr13MoS obtained by EDS: (**a**) sample was not corroded (Acceleration voltage: 20 kV, Working distance: 11.1 mm), (**b**) sample was corroded by ferric chloride hydrochloride solution (Acceleration voltage: 20 kV, Working distance: 9.9 mm).

EDS was carried out to investigate the distribution of various elements as shown in Figure 7a. The distribution of alloy elements Cr, Al, Ni, and Si is relatively uniform, while S, Mn, and Mo elements have obvious segregation, and these three elements gather in the same position, forming sulfides. It is known that MnS and MoS_2_ are formed according to the above analysis of Gibss formation energy. After corrosion as shown in Figure 7b, the self-lubricating phase forms many discontinuous holes, indicating that the corrosion resistance of the self-lubricating phase is poor. These corrosion holes destroy the continuity of the substrate, resulting in deterioration of the mechanical properties of the material. In addition, these heterogeneous phases are easy to become cavitation sources, accelerating the cavitation of parts. Therefore, it is necessary to study the morphology and size of these heterogeneous phases.

#### 3.2.2. Morphology and Size of These Heterogeneous Phases

**Figure 8:**
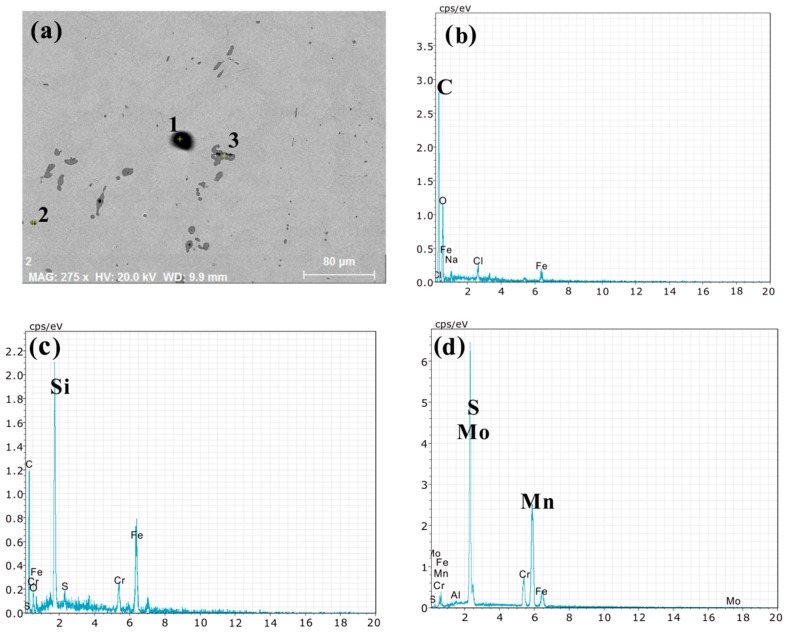
Heterogeneous phases in1Cr13MoS material, (**a**) SEM image of 1Cr13MoS material (Backscattered electrons, Acceleration voltage: 20 kV, Working distance: 9.9 mm), (**b**–**d**) correspond to the EDS of the inclusions at 1, 2, and 3 in Figure 8a, respectively.

The morphology and size of heterogeneous phase have a great influence on the properties of materials [17,18]. Figure 8a shows the morphology of some inclusions. Figure 8b–d show the composition of the inclusions at three places in Figure 8a respectively. According to Figure 8, there are some silicides and carbides except sulfides, which were generated during smelting, but here we focus on sulfides. MoS_2_ is mostly spherical in shape, with a few strips, a maximum length of 20 μm, a minimum of 2 μm, and an average size of about 6 μm, which improves the lubricity of the material, and has little effect on the strength of the material.

### 3.3. Friction and Wear Performance

**Figure 9:**
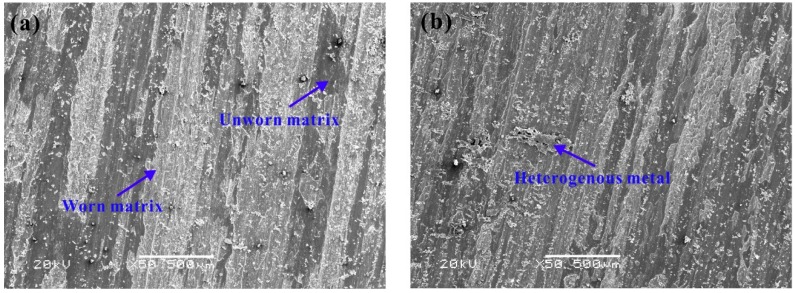
Morphology of blocks after friction test obtained by SEM (Secondary electrons, Acceleration voltage: 20 kV, Working distance: 10.0 mm), (**a**) X20Cr13, (**b**) 1Cr13MoS.

In the friction testing, X12Cr13 was used as a ring for both of two friction pairs, and X20Cr13 was used as block for such testing on the purpose of comparison. While X20Cr13 is a common material for using as wear ring in a pump. Both the two blocks have mass loss in the friction test as shown in Table 2. In the case that all other conditions are the same, the friction pair composed of 1Cr13MoS and X12Cr13 has lower wear and friction coefficient. The morphology of blocks was observed by SEM after the samples were washed and dried, and the results are shown in Figure 9.

The friction model can be regarded as a Hertzian contact process of a cylinder to a plane. In order to calculate the maximal contact pressure and the half-width of Hertzian contact by a simple method, Young modulus and Poisson ratio of X12Cr13, 1Cr13MoS, X20Cr13 were treated as the same value which were 203 GPa and 0.3 according to the ordinary stainless steel [19]. The maximal contact pressure and the half-width of Hertzian contact were calculated to be 39.73 Mpa and 0.014 mm respectively according to Equations (1) and (2) [20].

(1) $$\sigma_{\max}=0.418\sqrt{\frac{\mathrm{FE}}{\mathrm{lr}}}$$

(2) $$\text{b}=1.522\sqrt{\frac{\text{Fr}}{\text{lE}}}$$

where σ_max_ is the maximal contact pressure, F is the applied pressure, E is the Young modulus, l is the thickness of the ring, r is the radius of the ring. b is half-width of Hertzian contact. 

Results in Figure 9 show that there is obvious wear on the block surface of the two friction pairs, and there are thick traces of metal daubing on the friction blocks. Among them, the dark and smooth area is the block matrix metal material, and the white strip is the worn matrix. Compared with 20Cr13, 1Cr3MoS has a lower degree of surface tear and relatively light wear. In addition to obvious abrasive wear, a small amount of heterogeneous metal can be seen sticking to the block matrix, indicating that adhesive wear has also occurred. Since the hardness of the ring is lower than the hardness of the block, the base of the ring is more susceptible to wear and the surface fragments are torn and attached to the block substrate. Therefore, the heterogeneous phase as shown in Figure 9b is likely to come from the ring. Compared with 20Cr13, 1Cr13MoS has a higher wear-resisting layer, and the abrasive wear and adhesive wear coexist.

Due to the uncertain conditions in industrial applications, it is impossible to completely restore the operating conditions of the equipment. Our experimental results reflect the friction and wear properties of the material under such specific conditions. However, this also shows that the friction performance of 1Cr13MoS material is superior to the traditional X20Cr13 stainless steel. It is worth noting that the 1Cr13MoS is actually operated as friction pair material on the water pump and has a significant wear improvement effect compared to the conventional 12% chrome steel series.

## 4. Conclusions

The 1Cr13MoS stainless steel prepared by traditional pyrometallurgy has a self-lubricating performance due to the addition of S and Mo in the material. The self-lubricating phase MoS_2_ is discretely distributed on a steel substrate with an average size of 6 μm. The content of Mn needs to be strictly controlled to prevent a large amount of MnS from being generated to lower the self-lubricating property of the material. In addition, the hardness of 1Cr13MoS after annealed at 900 °C, quenched at 1000 °C, and tempered at 520 °C can reach HB 350 or above, and the tempered sorbite is uniform. It can be seen that the wear form of the friction pair composed of 1Cr13MoS material and X12Cr13 material on the block-on-ring tribometer is mainly abrasive wear and adhesive wear and the 1Cr13MoS material shows better friction and wear performance than the traditional X20Cr13 material through friction and wear experiments. The1Cr13MoS material has shown great application prospects on the friction pair of the pump.

## Figures and Tables

**Table 1:** Control range and measured value of the chemical composition of 1Cr13MoS (wt. %).

Element	C	Mn	Si	S	P	Cr	Ni	Mo	Fe
Control range	0.12~0.15	0.2~0.6	0.3~0.8	0.25~0.32	≤0.025	12~14	≤0.35	0.3~0.6	balance
Measured value	0.149	0.414	0.68	0.32	0.02	13	0.16	0.37	balance

**Table 2:** Results of friction test.

Friction Pairs		Mass Loss (g)	Friction Coefficient
Block	Ring	Block	
X20Cr13	X12Cr13	0.2954	0.8401
1Cr13MoS	X12Cr13	0.1665	0.5246
